# Factors associated with the life satisfaction amongst the rural elderly in Odisha, India

**DOI:** 10.1186/s12955-015-0398-y

**Published:** 2015-12-21

**Authors:** Pallavi Banjare, Rinshu Dwivedi, Jalandhar Pradhan

**Affiliations:** Department of Humanities and Social Sciences, National Institute of Technology, Rourkela, 769 008 Orissa India

**Keywords:** Elderly, Life satisfaction, Morbidity, Social support, Cognitive health

## Abstract

**Background:**

Life Satisfaction (LS) is an indicator of subjective well-being (SWB) among the elderly, and is directly associated with health and mortality. Present study deals with the factors associated with the LS among the rural elderly in Odisha, India.

**Methods:**

A cross-sectional survey using multi-stage random sampling procedure was conducted among elderly (60+ years) in Bargarh district of Odisha. The survey was conducted among 310 respondents. Hierarchical regression analysis was used to assess the adjusted effect of various socio-economic, demographic, health conditions (physical and mental), social support and effects of multi-morbidity on LS.

**Results:**

Cognitive health was the most influential factor in determining LS among both men (β = 0.327) and women (β = 0.329). Individual’s social support also plays an influential role in LS among rural elderly. Elderly who are living alone and have any sort of disability and had low score of activities of daily living (ADL) have also reported significantly lower perceived LS for both the genders.

**Conclusion:**

It is necessary to analyze and identify the major factors which can improve upon the level of LS among the elderly population. Better understanding of these factors can help in removing the superfluous anxiety of old age in the mindset of people which is pervading in the society.

## Background

Life satisfaction (LS) is an important component of successful aging. Successful aging is a universal phenomenon, which is not uniform across the different age groups, and it differs from person to person. Some accomplish a sense of fulfillment and satisfaction in old age, while others turn harsh and hostile to the changes of old age and lament on the decline of their physical activities [1, 2]. Level of LS indicates the subjective wellbeing which is associated with the health and mortality status among the elderly. It is among the one of the main determinants of well-being, which reflects the cognitive judgmental aspect of an individual [3]. Older people who experienced bad health tend to express low level of LS. However having higher socioeconomic status, adequate family support, higher level of satisfaction with one’s living environment/condition, and staying in their own home among the elderly population plays a crucial role in achieving successful aging [4, 5].

LS refer to a judgmental process, in which individual access their quality of life (QoL) in accordance with some unique set of criteria’s. Elderly are more sensitive to their LS and health conditions, which is further influenced by their socioeconomic situations, and limited work performance [6, 7].

As per the WHO, four factors which directly influences the level of LS among the elderly are: physical health condition, mental health condition, social relationship and environment [8]. For improving LS among the elderly, it is necessary to consider factors like satisfaction in residential environment, neighborhood relationship, economic status, maintaining friendship, family relationship, physical health condition, satisfaction in marital status, job or career, and lastly, satisfaction in others aspect of life [9].

LS is influenced by various factors like demographic, socio-economic, health, physical status, mental status, social support, social adjustment and number of morbidities. Studies indicate that factors such as race, socioeconomic status, marital status, education, level of self-esteem, depression, may influence the level of LS. In terms of demographic factors, increase in age has a significant impact on the LS among females in comparison to males [10]. Few studies have recorded that neither age nor gender was positively associated to LS [11, 12]. Time-varying physical health is related to changes in LS [13]. Change in marital status is related to the LS path, and new widowhood is related to morale and social engagement. Women and men in formal marriages experience higher levels of LS than people in other forms of marriages [14, 15].

There is a moderate effect of socioeconomic status, including income and educational level, on LS [16–19]. A study on LS amongst elderly people living in Australia, found that social, health, security of life, residence, acceptance and adjustment influences the level of LS among the elderly [20]. Studies also showed that residential status have a negligible impact on LS [21].

Most of the research was focused on activities of daily living (ADLs) and instrumental activities of daily living (IADLs) as an indicators to evaluate the health condition of elderly and LS. Physical activities, viz. bathing, dressing, toileting, continence, movement and food intake including ADL, are positively related to LS [22, 23]. However, few studies show that physical disability was not significantly related to LS [24]. As per a study among 132 countries, by Gallup organization, there are stronger evidences that health and LS among the elderly people decreases with increase in the level of disability and age. This study also indicates towards differences among the developed and developing countries as decline in LS with disability and age was more among the developing and underdeveloped nations [25].

There is a positive relationship between psychological well-being and LS. Psychological health is generally related to overall subjective well-being, and there is a significant relationship between depressive symptoms and LS [26, 27]. Psychosocial variables like size of social support, social support and positive social relations are strongly related to LS [28, 29]. There is a significant effect of physical and psychological well-being on LS while, socio-demographic variables such as gender or age plays very limited role in LS [30, 31].

Social support systems like religion, education, marriage, occupation, active daily life status, living arrangement, diet, transportation, family support and emotional support also have a positive impacts on the LS of elderly [32, 33]. If the income of the households is lower in that case family support has a greater effect on happiness among elderly while emotional support has been found to work as a shield during traumatic periods and increases LS [34]. In few communities, relationships with friends are more important for the LS among elderly population [35]. Continuous working and participation in volunteering activities or community events are also related to the higher level of subjective well-being [36].

Various studies indicate social support as a factor which has a strong influence on the LS among the elderly. Literature suggests that psychological variable and locus of control affects subjective well-being more than social support and LS in elderly individuals [37]. Factors like household environment (both physical and social), overall well-being, personal information (information on age, sex, education and place of residence), self-acceptance (social support), personal traits (cognitive health) and lifestyle indicators (smoking, consuming tobacco and alcohol) are correlated with the LS among the elderly population [38, 39].

Studies also explored the factors associated with the LS which have been experienced during the process of aging. In case of within the group distribution of elderly population (health inequity)^1^, results indicates that economic position and health were the most important factors determining levels of LS among elderly individuals [40]. LS is a forecaster of longevity and morbidity, disease and injury [24]. In addition, LS is also related to other health variables such as favorable self-reported health, social support, and positive health behaviours. Limited evidences are available on the relationship between LS, health behaviours, chronic health conditions, and health-related quality of life (HRQOL) among the elderly. Behavioural Risk Factor (smoking, consumption of alcohol and tobacco) chronic illness and adverse health behaviours are correlated with HRQOL and influences LS [41–43].

In Indian context, Maheswaran and Ranjit [44] focused on LS and influence of demographic factors on LS of the elderly people. Results indicate that majority of the respondents had low level of LS. Moreover, the demographic factors namely gender and habit of savings directly influences the level of LS of the elderly respondents. The variables viz. health problems, ownership of house, ownership of land, religion, monthly income and number of children, negligibly influence the level of LS.

Balachandran et al. [45] studied LS and alienation of elderly males and females in the district of Kerala, India. The results indicate that elderly men experience less alienation in comparison to the elderly women. However, both elderly male and females do not exhibit significant differences in their LS. Marpady et al. [46] conducted a study in rural Karnataka, India to explore the pattern of social support system and LS amongst the elders. The research revealed that family support is a significant factor for the better psychological wellbeing of the elderly. It was also observed that homebound elderly had more advantages than the institutionalized elderly in terms of daily activities, level of satisfaction, social support and source of financial support.

India is the second most populated country in the world, with nearly 100 million persons aged above 60 years, which constitutes about 8.3 % share in the total population [47]. As the elderly population is on a rise throughout the globe specifically in India, various aspects of ageing and its precursors have become an area of research for gerontologists and social scientist.

After reviewing the literature from international as well as in Indian context, major gaps has been identified. Limited number of studies has highlighted the role of either demographic, socio-economic, social support or cognitive health. As per our information there are no such studies which have completely addressed the issue of LS among the rural elderly by taking into consideration all major variables which influences the level of LS. Present study is an attempt to fill this existing gap in the literature and provides a linkage between all the major factors such as demographic, socio-economic, social support and cognitive health variables. This paper examines the relationship between various covariates of LS among the rural elderly in Bargarh district of Odisha on the basis of following hypothesis:

- H1: Elderly having better cognitive health are having better LS.
- H2: Elderly having good social support are having better LS.
- H3: Elderly having no morbidities are having better LS.

This paper aims to analysis the factors constructing LS amongst the rural elderly of Odisha and explores its implications on their health and life, which can positively contribute in developing social policies.

## Methods

### Ethics statement

The study was conducted in Bargarh district of Odisha, India. The study aims to explore the familial setups, roles, health status and expectations of the elderly. Before collecting necessary information from selected elderly, following consent form was signed by the respective respondent:

*“I am going to ask you some personal questions that some of the people find difficult to answer. Your answers are completely confidential, your name, will not be disclosed to anyone, and will never be used in connection with any of the information you tell me. You do not have to answer any questions that you do not feel comfortable, and you may withdraw from this interview at any time you want to. However, your answers to these questions will help us to understand the senior citizens situation. We would greatly appreciate your help in responding to this interview. Would you be willing to participate?”*

If the respondent provided consent, an interview was conducted.

The study was approved by the Doctoral Research Committee (DRC) of National Institute of Technology, Rourkela, Odisha, India.

### Study area and subjects

**Fig. 1:**
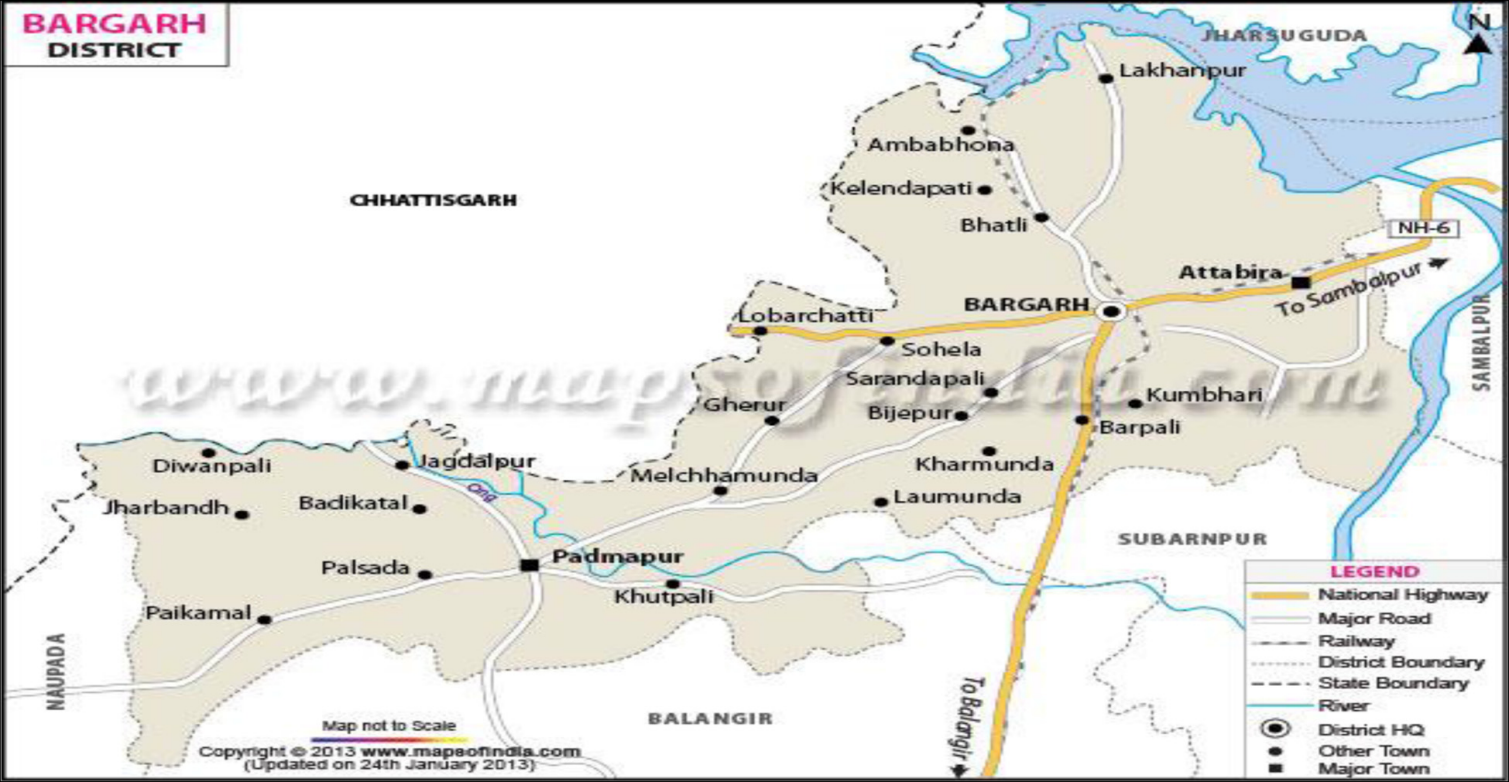
Map of Bargarh district in Odisha

A cross-sectional survey using multi-stage random sampling procedure was conducted among elderly (60+ years) in Bargarh District of Odisha (Fig. 1). The targeted sample size of the population was 320. Data were collected by face-to-face interviews with a pre-tested structured questionnaire. Ten respondents who were extremely frail could not respond to the questionnaires. So, finally 310 respondents were considered for analysis resulting in a response rate of 97 %. In order to increase the efficiency of estimates, a multistage sampling design was followed to select required number of respondents for interview. Selection of respondents involved three stages of sampling procedure. Block 2 was selected at the first stage. Then village^3^ was selected at the second stage followed by selection of target respondents at the third stage.

As per Census 2001, there are 12 blocks in Bargarh i.e. Bargarh, Barpali, Attabira, Bheden, Sohella, Bijepur, Padmpur, Gaisilet, Paikmal, Jharbandh, Ambabhona and Bhatli. Two blocks namely Sohella and Padampur were selected randomly. Twenty respondents (10 Male and 10 Female) were selected from each village. So, 16 villages (8 from Sohela and 8 from Padampur) were selected to get the required number of respondents. Villages were selected using probability proportion to sample size (PPS). At the village level, a sampling framework was prepared separately for male and female respondents. A complete listing of the households in a selected village was done. During the listing in each household all the members aged 60+ were listed. Each member’s actual age and gender were noted. Accordingly, 10 Male and 10 Female elderly were selected by using systematic random sampling procedure. The study was conducted in Bargarh district as the percentage of elderly population was higher than the state average and ratio of female elderly population was also higher in the district than the male elderly population [47].

### Framework of the study

**Fig. 2:**
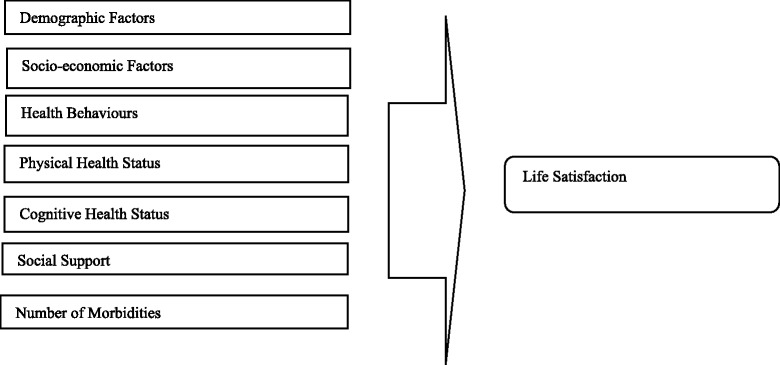
Framework of this study model by (Coke and Twaite (1995), Naugarten et al.(1965))

In order to investigate various factors associated with LS among elderly we have partially derived the framework of the study from Coke and Twaite [48] and Neugarten et al. [49]. As per Life Satisfaction Index (LSI), successful ageing depends upon the general feelings of well–being among older people and positively contributes into the LS. The concept of LS is associated with various factors like demographic variable, socio-economic factors, health behavior, physical health status, cognitive health status, social support and number of morbidities among elderly**.** We have recorded responses on these above mention aspects by asking questions through a structured questionnaire from the respondents. The framework of the study is summarized in Fig. 2.

### Katz Index of independence in ADL

As aging process sets in it results into the changes in the health status of the individual and decline in the functional status of older adults. The most appropriate method or tool to access ADL of the elderly is the Katz Index of Independence in ADL, commonly referred to as the Katz ADL. It was developed by a medical doctor Sidney Katz In 1969 [50]. The Index ranks adequacy of performance in the six functions of bathing, dressing, toileting, transferring, continence, and feeding. Clients are scored yes/no for independence in each of the six functions. A score of 6 indicates full function, 4 indicate moderate impairment, and 2 or less indicates severe functional impairment.

### Variables under study

Variables used for the study (both dependent and independent) has been explained below.

- Dependent variables: We have taken LS as our dependent variable. In order to determine LS, respondents were asked, “Taking all things together, how would you say you are these days”? The responses were on Likert scale ranging from (1.very happy, 2.happy, 3. neither happy nor unhappy, 4. unhappy and 5. very unhappy.
- Independent variables: The independent variables are; a) Age (reference group: 65+ years), b) Marital status (reference group: Not currently married), c) Caste^4^ (reference group: SC/ST/ general), d) Education (Likert scale) e) Wealth quintile (Likert scale), f) state of economic independence (reference group: dependent), g) living arrangements (reference group :living alone), h) Risk behaviour (reference group: consuming tobacco, smoking, and drinking alcohol i) disability status (reference group: not disabled), j) Functionality or ADL (reference group: functional), (k) cognitive health status (reference group: bad cognitive health, l) social supports (reference group: not having good social supports), m) morbidity status (reference group: no morbidity). We tested multi-co linearity for all independent variables, and in no case was the tolerance value less than 0.1 or the variance inflation factor (IVF) greater than 10 (Table 1).

**Table 1:** Variables used in the study

Predictive variables	Parameters	Instruments
Health Behaviours	Question : Do you have any of the following habits	Questionnaire containing dichotomous responses (*Yes/No)*
	a) Smoking	
	b) Consumption of alcohol	
	c) Consuming tobacco	
Social networks	Question: Do you have keep in touch with your relatives or friends?	Questionnaire containing dichotomous responses (*Yes/No)*
Morbidity	No Morbidity	International Classification of Diseases (ICD)-10
	At least one morbidity	
	At least two morbidity	
	Three or more morbidity	
Disability		
Physical disability	Vision, hearing, walking, chewing, speech, memory.	Questionnaire containing dichotomous responses (*Yes/No*)
Activities of Daly Living (ADL)	Feeding, continence, transferring, toileting, dressing, bathing.	Katz scale
Psychological distress	12 questions related with psychological wellbeing among elderly	General Health Questionnaire (GHQ)-12

### Statistical analysis

Statistical analysis was carried out in two stages: firstly; the sample characteristics of the elderly population was assessed for each variable using descriptive statistics by also focusing upon the observation of elderly population with chronic diseases. Later on, hierarchal multiple regression analysis was used as per Gender. Hierarchal regression by gender was employed as per the available literature which shows that gender has a significant effect on LS among elderly. Literature on differences in pain experiences indicates towards gender variations, where women generally experience more experimental pain in comparison to men. Moreover the perception, attitude and approach towards life are very different among both the genders. Level of LS is lower among those men who are living alone or without their spouse while among females, LS is lower when they used to stay with their relatives or their parents-in-law. Similarly residing with an unmarried son is negatively associated with LS for both genders [51]. Women LS increases with higher number of social activities and friend circle which is not that significant predictor of LS among the males [52]. Overall family relations are of more importance to males in comparison to females. Elderly men whose marital status remained stable there LS was also constant while in case of females there was a decline in LS. In addition, among males LS increases with marriage while it has no significant role for females [53]. Data analysis was done using SPSS 20 software.

## Results

The sample characteristics of the studied population by selected socio-economic covariates shows that out of the total sample of 310 respondents, 153 are male and 157 are female (Table 2). The married people comprise of 60.3 % and widowed / divorced or separated comprise of 39.7 % of the total sample. Study on Literacy or Education of the respondent’s shows that about 60.3 % have no formal education, followed by 27.7 % who have completed primary education or less and only 4.5 % have completed their secondary school and above. In State of Economic Dependence, about 46.5 % are partially dependent, followed by not dependent on others and 11.3 % are fully dependent on their spouse, son or other relative. While analyzing caste structure, Other Backward Caste (OBC) have the highest share of 57.1 %, followed by Scheduled Caste (SC)/ Scheduled Tribe (ST) with 31.9 % and general have 11 % only. Elderly living with spouse and married son are about 54.5 %, followed by living with either spouse/son or daughter and elderly living alone are the least with only 7.7 % share. Predominantly, in the to the Indian system of old-age support and living arrangement there is the norm that sons support their parents; that is, they provide financial and practical support for their elderly parents. Elderly parents mostly live with their elder son and daughters are treated as outsiders after their marriage [54] Staying with a married daughter is still regarded as a taboo in rural settings in India. Those elderly who don’t have any son stay either alone or with some relative and daughters often visit them and provide them with some financial support [55].

**Table 2:** Percent distributions of sampled population by selected socio-economic co-variates, health conditions and risk behaviour

Covariates	Percent	Number
Sex		
Male	49.4	153
Female	50.6	157
Age of the respondents		
60–65 Years	30.6	95
65–70 Years	35.5	110
70–75 Years	20.0	62
75 & Above	13.9	43
Marital Status		
Currently married	60.3	187
Widowed/Divorced or Separated/Never married	39.7	123
Education status of respondents		
No formal education	60.3	187
Less than primary	27.7	86
Primary school completed	7.4	23
Secondary school and above	4.5	14
Wealth quintile		
Poorest	19.7	61
Poorer	19.4	60
Middle	21.0	65
Richer	19.7	61
Richest	20.3	63
Caste		
General	11.0	34
Scheduled Caste/Scheduled Tribe	31.9	99
Other Backward Caste	57.1	177
State of economic dependence		
Not dependent	42.3	131
Fully dependent	11.3	35
Partially dependent	46.5	144
Living arrangements		
Living alone	7.7	24
Living with spouse/Son/Daughter	25.5	79
Living with Spouse and unmarried son	12.3	38
Living with Spouse and married son	54.5	169
Morbidities		
No morbidity	10.3	32
Having one morbidity	32.9	102
Having two morbidity	26.8	83
Having three or more morbidities	30.0	93
Risk Behaviours		
Smoking (Yes)	31.0	96
Consuming Alcohol (Yes)	4.19	13
Consuming Tobacco (Yes)	63.2	196
Disability		
Disabled	29.4	91
Not Disabled	70.6	219
Social support		
Yes, keep in touch with relatives	63.2	196
No, Don’t keep in touch with relatives	36.8	144
Cognitive health		
Poor	49.7	154
Average	36.1	112
Good	14.2	44
Functionally or Activities of daily living (ADL)		
Functional	76.8	238
Not functional	23.2	72
N	100	310

As this study was also done in the rural setup the percentage of elderly living with the married daughter was negligible so it was not taken into account. About 58.1 % of the population have Below Poverty Line (BPL) card. About 63 % of the respondents are consuming tobacco, 31 % of them are used to smoking and a small proportion (4 %) in drinking alcohol. Nearly 63 % of the elderly are having good social support. Majority of the elderly were not disabled (70 %) as only 30 % of them were having some sort of disability. Half of the elderly population was having poor cognitive health status and only 14 % of the elderly were having good cognitive health status. About 77 % of the elderly population in the district was in functional state and 23 % have some issues with ADL.

While comparing the prevalence of disease amongst males and females, it shows that arthritis is more common among females than males, whereas Chronic Obstructive Pulmonary Disease (COPD) and high blood pressure are more common among males. Similarly, dementia and Alzheimer’s disease are more common among females and cataract amongst males. For other diseases, both male and females shared similar patterns with few variations (Table 3).

**Table 3:** Percent of respondents having selected morbidities by Gender

Morbidities	Male (*N* = 153)	Female (*N* = 157)	Total (310)
Arthritis	50.9	54.7	52.9
Cerebral-embolism, stroke or Thrombosis	0.6	1.9	1.2
Heart disease	0.6	4.4	2.5
Diabetes	7.8	10.8	9.3
Chronic obstructive pulmonary disease	30.0	10.1	20.0
Asthma	9.1	10.1	9.6
Depression	7.1	4.4	5.8
High blood pressure	26.1	12.7	19.3
Alzheimer’s disease	3.9	9.5	6.6
Cancer	0.0	1.9	0.9
Dementia	4.5	7.6	6.1
Liver or gall bladder illness	4.5	3.1	3.8
Osteoporosis	1.9	3.1	2.5
Renal or Urinary tract infection	9.1	3.8	6.4
Cataract	21.5	15.9	18.7
Loss of all natural teeth’s	4.5	7.0	5.8
Accidental injury (in past one year)	11.7	6.3	9.0
Injury due to fall (in past one year)	3.9	2.5	3.2
Skin disease	6.5	7.0	6.6
Paralysis	8.4	4.4	6.4

**Fig. 3:**
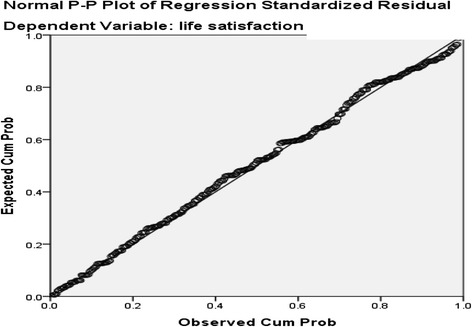
Normal P-P Plot of regression standardized residual of life satisfaction

**Fig. 4:**
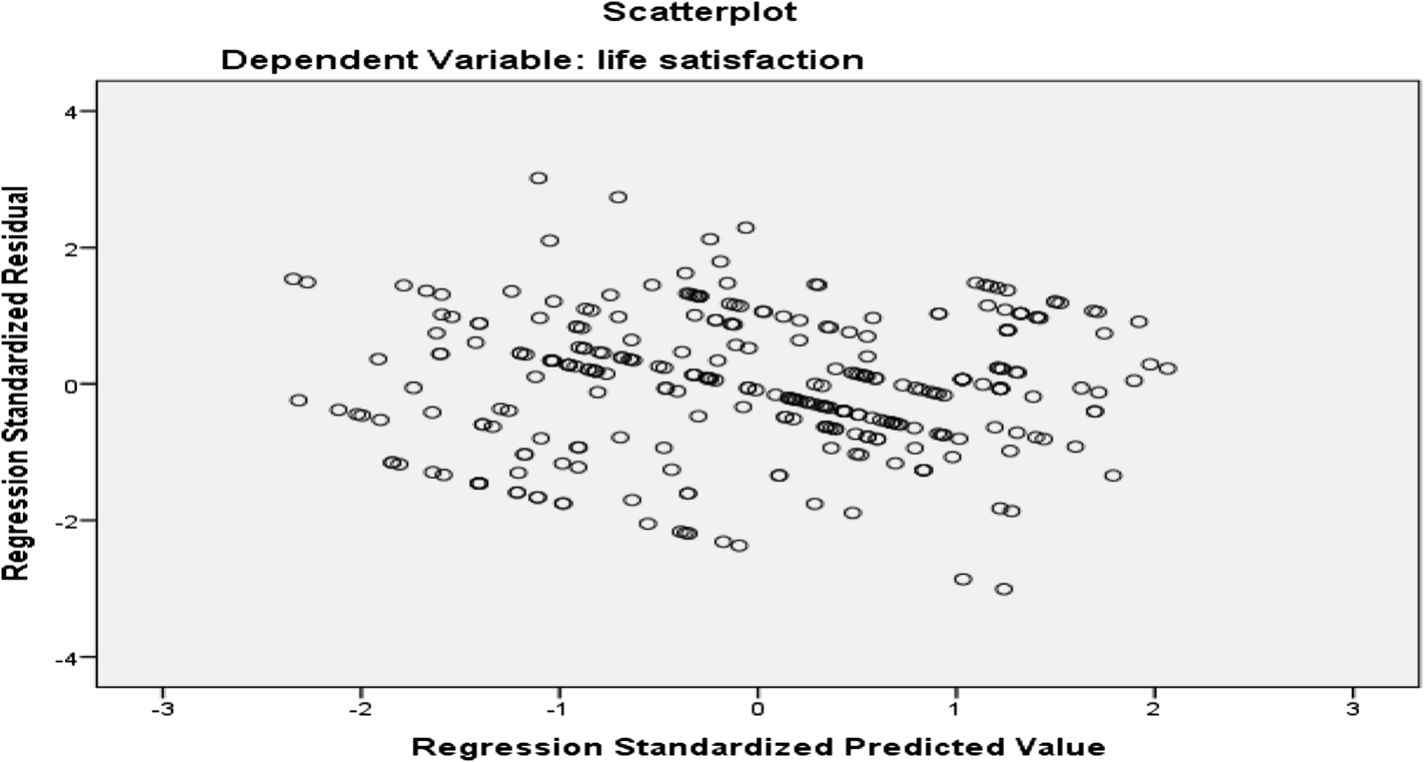
Scatter plot of life satisfaction among elderly population

The normal P-P plot of regression standardized residual and scatter plot, indicates the outliers, normality, linearity and homoscedasticity. The purpose of P-P plots is to check if the data are normally distributed so here the data are plotted against a theoretical normal distribution in such a way that the points should form an approximate straight line. Departures from this straight line indicate departures from normality. Here the normal P-P plot points are lying in the straight diagonal line from the bottom left to top right showing normal distribution of data and fit for analysis. In scatter plot, residuals are rectangularlly distributed with most the scores concentrated at the center i.e. along the point O. Standardized residuals of more than 3.3 or less than −3.3 indicates outliers (Figs. 3 and 4) [56].

The model summary of both dependent and independent variables shows *R*^*2*^value and variables entered in Block one (age, caste, marital status, education, wealth index, state of economic dependence, living arrangements, life style indicators, ADL, cognitive health and social adjustment,). They together explain 30 % of the variance (.30*100) in dependent variable (DV). After entering the variable gender in block two the model now explains 35 % of variance in the DV. The *R*^*2*^change in model two here again explains an additional of 5 % variance in DV by variable gender. This is a significant contribution, as indicated by *sig F* change value for this line (0.000) (Table 4).

**Table 4:** Model summary of LS

Model Summary^**c**^									
Model	R	R Square	Adjusted R Square	Std. Error of the Estimate	Change Statistics				
					R Square Change	F Change	df1	df2	Sig. F Change
1	.552^a^	.304	.266	3.498	.304	8.014	16	293	.000
2	.583^b^	.350	.302	3.513	.086	15.796	1	292	.000

a*predictors: (constant) age, caste, marital status, education, wealth index, state of economic dependence, living arrangements, life style indicators, activities of daily living, cognitive healh and social adjustment

b*predictors: (constant) age, caste, marital status, education, wealth index, state of economic dependence, living arrangements, life style indicators, activities of daily living, cognitive health and social adjustment, gender

c*dependent variable: life satisfaction

Present method is suitable for this study as in this method the effects and coefficient values of each factor on the level of LS and incremental change (increase/decrease) in the *R*^*2*^value are provided in comparable forms. All the five models are considered for the analysis and variables are added in each model and the final model contains all the 13 variables used in the analysis (Table 5). We have conducted a hierarchical regression analysis with respect to gender in order to identify the main covariates which determine the level of LS in elderly population (Tables 6 and 7). Five sets of models were introduced and the result of final model is presented after the introduction of 13 variables. Results shows that 6 variables were statistically significant for both men (adj. R^2^ = 0.384) and women (adj. R^2^ = 0.386).

**Table 5:** Model design for regression analysis

Models	Model 1	Model 2	Model 3	Model 4	Model 5
Variables	Only demographic variables	Only Socio-economic variables	Only life style indicators	All independent covariates	All independent covariates
	• Age	• Education	• State of economic dependence	• ADL (activities of daily living)	• Age
	• Marital status	• Wealth Index	• Living arrangements	• Social supports	• Marital status
		• Caste	• Risk behaviour (Smoking, Consuming tobacco and alcohol)		• Education
					• Wealth quintile
					• Caste
					• State of economic dependence
					• Living arrangement
					• Risk behaviour (Smoking, Consuming tobacco and alcohol)
					• ADL (activities of daily living)
					• Social supports
					• Disability
					• Cognitive health
					• Morbidity status

**Table 6:** Model design for multiple linear regression for LS for males

Variables	Model I	Model II	Model III	Model IV	Model V
Age of the respondents^b^	0.003	0.005	0.004	0.015	0.019
Marital status^b^	0.936	0.084	0.007	0.004	-0.023
Education^a^		-0.02	-0.032	-0.007	-0.008
Wealth quintile^a^		0.196	0.136	0.125	0.069
Castle^b^		0.009	-0.023	-0.053	0.052
State of economic dependence^b^			0.044	0.044	0.074
Living arrangements^b^			0.165**	0.161	0.179
Risk behaviours					
a) Smoking^b^			0.029	0.061	0.102
b) Consumption of alcohol^b^			0.3	0.28	0.357
c) Consuming tobacco^b^			0.109	0.162	0.068
Activities of daily living (ADL)^b^				-0.546*	-0.287*
Social supports^b^				0.192**	0.286**
Disability^b^					-0.479**
Cognitive health^b^					0.327*
MOrbidity status^b^					-0.077
Adjusted R^2^	0.002	0.116	0.155	0.244	0.384
Change in Adjusted R^2^		0.114	0.039	0.089	0.140

** ρ <0.05; * ρ <0.01

Dependent Variable: Likert scale (1.very happy, 2.happy, 3. neither happy nor unhappy, 4. unhappy, and 5. very unhappy

The suggested figures in all cells are standardized B

^a^Continous variables: 5-point Likert scale

^b^Binary variables

**Table 7:** Model design for multiple linear regression for LS for females

Variables	Model I	Model II	Model III	Model IV	Model V
Age of the respondents^b^	0.002	0.005	0.005	0.016	0.02
Marital status^b^	0.905	0.091	0.067	-0.003	-0.031
Education^a^		0.026	-0.025	-0.001	-0.002
Wealth quintile^a^		0.196	0.136	0.125	0.068
Castle^+^		0.008	-0.02	-0.051	0.054
State of economic dependence^b^			0.041	0.041	0.072
Living arrangements^b^			0.164**	0.16*	0.178
Risk behaviours					
a) Smoking^b^			0.025	0.57	0.098
b) Consumption of alcohol^b^			0.283	0.264	0.342
c) Consuming tobacco^b^			0.109	0.162	0.17
Activities of daily living (ADL)^b^				-0.548**	-0.288*
Social supports^b^				0.189*	0.284**
Disability^b^					-0.476*
Cognitive health^b^					0.329**
MOrbidity status^b^					-0.074
Adjusted R^2^	0.004	0.119	0.159	0.246	0.386
Change in Adjusted R^2^		0.115	0.040	0.087	0.140

** ρ <0.05;* ρ <0.01

Dependent Variable: Likert scale (1.very happy, 2.happy, 3. neither happy nor unhappy, 4. unhappy, and 5. very unhappy

The suggested figures in all cells are standardized B

^a^Continous variables: 5-point Likert scale

^b^Binary variables

The most important factor that was influencing LS for both males and females are: individual’s cognitive health,(β value; M = 0.327, F = 0.329);social support (β value; M = 0.286, F = 0.284); living arrangements (β value; M = 0.179, F = 0.178); disability (β value; M = −0.479, F = −0.476); ADL or functionality (β value; M = −0.286, F = −0.284) and, morbidity status (β value; M = −0.077, F = −0.074). The observed differences in the results among men and women can be summarized as follows:

In model V there has been highest increase in the explanatory power of the *R*^2^ value in terms of LS for both men and women. We have further added variable like disability, cognitive health and number of morbidities in model V due to the explanatory power of *R*^2^ was measured at 0.140 in both male and female. Second highest increase in the explanatory power of *R*^2^ was observed in model IV where the change in adjusted *R*^2^ value increased to 0.089 in men and 0.087 in women. In this model, the variables introduced were ADL and social support.

## Discussion

Disability has a significant effect on the LS of elderly people. It is also observed that negative life events may increase psychological distress among elderly people and in turn can lower the level of LS. However there is a direct relationship between social supports and LS, as social support increases LS also increases. Furthermore, disability, preceding psychological distress, lack of friend circle or social support system that could help elderly, low ADL and IADL scores was associated with dissatisfaction with life or lower level of LS [57, 58].

LS and mental health are associated with some specific demographic factors such as age and gender. In addition, self-rated health and limited functionality due to disability exert a significant impact on psychological well-being and can lead to depressive symptoms, and psychological distress. The relationship between ADL, psychological factors and LS was different between males and females. It is a well-established fact that with increasing age, there are higher likelihood of morbidity and disability. This may be due to the nature or occurrence of disease as older people do not encounter with fatal diseases rather they suffer from chronic diseases [59, 60].

The LS of the elderly population relies on factors such as living arrangement, health condition, economic status, social support and financial status of their children. In case if the elderly people are having similar mind set as of their children, they can enjoy a healthier and happier life span with them. Moreover if they have enough financial resources to meet their basic needs and have adequate social support then they have higher LS. Living alone results into lower LS than those elderly who are living with spouse or their children [61, 62].

Our results conclude that most important variable which affects the level of LS of the rural elderly is cognitive health status. In this study, majority of the sampled elderly (50 %) were recorded with bad cognitive health. Elderly having low GHQ −12 score are prone to depression leading to lower LS [29]. The relationship between cognitive health status and LS is very crucial which can be assessed from the individuals' overall cognitive assessment of their living conditions and achievements, comparing them to their needs and expectations and in light of their personal and socio-cultural values [63]. The second most influential factor on LS is social support. Many studies have reported that social activities or interpersonal relationship enhances both physical and mental health. It lowers mortality rates among elderly [61, 62]. Social supports improves one’s self esteem, sense of belongingness and gives a purpose to life. It also provides physiological benefits such as better immune functioning and increased cardiovascular activities. It also promotes positive health behaviour like proper diet, exercise and helps a person to stay away from risky behaviour such as smoking and consumption of alcohol [64]. However the degree of social supports varies with the disability and morbidity status of an individual [65]. The next important factor effecting LS among rural elderly was morbidity status. There was an inverse relationship between the morbidity status of a person and his LS. Good health allows the person to maintain social contacts, resulting in higher level of LS and prevalence of chronic diseases results into low level of LS. Low LS is associated with ADL and IADL which is necessary for overall QoL. Chronic conditions like diabetes, hypertension and cardiovascular diseases have adverse effect on the health and it’s harmful for both cognitive and physical disabilities [66]. The other important variable for LS for rural elderly was living arrangements. In our study more than half of the elderly were living with spouse and married son. Elderly living with family members showed increased LS than those living alone or with relatives. Elderly living alone are unable to meet their basic requirements in case of disability and chronic conditions. Having support from family members reduces the effect of diseases and it gives them will power and strong mental attitude to manage their day to day life. Living arrangements influences the cognitive health and level of LS. Loneliness is a biggest enemy in old age which pushes a person towards morbidity leading to disability [67–69]. Living arrangements acts as a powerful instrument in defining roles among elderly as it gives a sense of belongingness and provides social support both formal and informal [70–72] . Spouse can be an important support for both male and females as the males can looks after for finances and females take care of health issues [53].

### Limitations of the study

It was a major challenge to explain to the respondents and their family members about the reason behind this study and the underlying benefit to them. Many respondents were curious to know whether they will receive any financial benefit from the government or not. This is a cross sectional study and results may change over time. This study takes into consideration only self-reported cases for any sort of morbidity and disability. No clinical examination has been performed, so the results may vary. The study is confined to few villages of Bargarh district in Odisha. It can be extended to other districts also to provide a probable solution to the problems faced by elderly that can be useful for the decision makers in future for policy implications.

## Conclusions

In this study, the subjective meaning of ageing is determined on the basis of the LS model. Factors such as cognitive status, morbidity status, and social supports can be the areas of concern and special focus for the gerontologist. Interdisciplinary research with the aim of increasing LS in the elderly should be promoted. Management of health conditions and ADL, IADL functioning plays a pivotal role in increasing LS among elderly. The issue of cognitive wellbeing remained unidentified especially among the elderly population. This issue needs more attention as sound cognitive health can enhance the overall LS among the elderly. Our study brings into focus this unattended issue of cognitive health which can be looked into by the policy makers. Proper designing of the welfare programmes, policies and regulations for elderly needs a better understanding of the relationship between people’s individual characteristics and their perceptions regarding LS. There should be well trained staff and infrastructure in the geriatric units to cater the cognitive needs of the rural elderly which is fully ignored at present in India. In the Indian context social support system is essential for ensuring psychological wellbeing among the elderly population. More stress should be laid upon the counseling among the younger generation which can encourage them to support and look after the needs of their elderly as it will enhance the level of LS. There should be more focus in building intergenerational solidarity among the two generations which will improve the social support. Disabled elderly should be given special attention by the society and more user-friendly environment should be provided to them. This study can contribute positively in the overall feeling of happiness among the elderly population which may become a basis for the formulation of policies to improve the QoL in the county like India. The implication of our findings can be adopted by the government agencies and police maker to identify the major areas of attention and to identify the most vulnerable aged people (70 above), particularly aged females/widows.

## Abbreviations

ADL: Activities of daily living
BPL: Below Poverty Line
COPD: Chronic Obstructive Pulmonary Disease
DV: Dependent variable
HRQOL: Health-Related Quality of Life
IADL: Instrumental activities of daily living
IV: Independent variable
LoC: Locus of control
LS: Life satisfaction
OBC: Other Backward Caste
PPS: Probability proportion to sample size
QoL: Quality of life
SC: Scheduled Caste
SEM: Structural Equation Modeling
ST: Scheduled Tribe
SWB: Subjective wellbeing

## Footnotes

1 Health inequity refers to the unfair distribution of health determinants, outcomes and resources within and between segments of the population, regardless of social standing (CDC, 2010).

2 Territorial or administrative units from the hierarchy of census, India.

3 A village is a clustered human settlement or community, larger than a hamlet but smaller than a town, where population ranges between few hundred to a few thousand.

4 The caste system in India is a closed system of social stratification with its origin in pre-modern era that uses to determine the access to wealth power and privilege in India.
